# Progranulin acts in lysosomes to promote neuronal survival and growth

**DOI:** 10.1002/alz.093001

**Published:** 2025-01-03

**Authors:** Azariah K Kaplelach, Justin A Hall, Skylar E. Davis, Jonathan R Roth, Qays Aljabi, Ahmad R. Hakim, Katherine Savell, Jeremy J Day, Andrew E. Arrant

**Affiliations:** ^1^ University of Alabama at Birmingham, Birmingham, AL USA

## Abstract

Progranulin is a secreted pro‐protein that is necessary for maintaining lysosomal function and exerts anti‐inflammatory and neurotrophic effects in the brain. Loss‐of‐function *GRN* mutations, most of which cause progranulin haploinsufficiency, are a major autosomal dominant cause of frontotemporal dementia (FTD). Other *GRN* variants are associated with risk for FTD, Alzheimer’s disease (AD) and Parkinson’s disease. A common AD‐ and FTD‐associated *GRN* variant is also associated with mild reduction of progranulin levels. Thus, loss of progranulin’s lysosomal, anti‐inflammatory, and/or neurotrophic effects may underlie the association of *GRN* with neurodegenerative disease. Restoring progranulin is a promising treatment strategy for people with *GRN* mutations and perhaps for a broader group of patients. Understanding progranulin’s mechanism of action could enable design of optimal progranulin‐based therapies. Progranulin is constitutively secreted and can interact with several signaling receptors before being taken up and trafficked to lysosomes. It is not clear if progranulin’s anti‐inflammatory and neurotrophic effects are mediated by extracellular signaling or actions in lysosomes. To distinguish between these mechanisms, we developed lentiviral vectors expressing human progranulin (PGRN) fused to the transmembrane domain and cytosolic tail of LAMP‐1 (L‐PGRN). We used these vectors in mixed primary neuron/astrocyte cultures to test the hypothesis that progranulin’s neurotrophic effects are mediated in lysosomes. L‐PGRN was not secreted, but was trafficked to lysosomes, cleaved into granulins, and able to normalize lysosomal enzyme activity in *Grn^–/–^* neurons. L‐PGRN mimicked the protective effects of PGRN against NMDA excitotoxicity in primary cortical neurons. Delivery of L‐PGRN with neuron‐specific vectors confirmed that this effect was mediated directly in neuronal lysosomes. Further investigation revealed that L‐PGRN blocked the contribution of autophagy to excitotoxic cell death. L‐PGRN was also as effective as PGRN at promoting dendritic outgrowth in primary hippocampal neurons. A neuron‐specific L‐PGRN vector failed to promote neuronal outgrowth, but an astrocyte‐specific L‐PGRN vector promoted neuronal outgrowth similarly to the non‐specific L‐PGRN vector. Progranulin may therefore promote dendritic outgrowth through a non‐cell–autonomous mechanism involving astrocytes. We are currently investigating how progranulin may act in astrocytic lysosomes to change reactive and/or neurotrophic phenotypes.

